# Detecting Reconnaissance and Discovery Tactics from the MITRE ATT&CK Framework in Zeek Conn Logs Using Spark’s Machine Learning in the Big Data Framework

**DOI:** 10.3390/s22207999

**Published:** 2022-10-20

**Authors:** Sikha Bagui, Dustin Mink, Subhash Bagui, Tirthankar Ghosh, Tom McElroy, Esteban Paredes, Nithisha Khasnavis, Russell Plenkers

**Affiliations:** 1 Department of Computer Science, University of West Florida, Pensacola, FL 32514, USA; 2 Department of Mathematics and Statistics, University of West Florida, Pensacola, FL 32514, USA

**Keywords:** Apache Spark, big data, network traffic analysis, intrusion detection systems, machine learning, Zeek Connection Logs, MITRE ATT&CK^®^ framework

## Abstract

While computer networks and the massive amount of communication taking place on these networks grow, the amount of damage that can be done by network intrusions grows in tandem. The need is for an effective and scalable intrusion detection system (IDS) to address these potential damages that come with the growth of these networks. A great deal of contemporary research on near real-time IDS focuses on applying machine learning classifiers to labeled network intrusion datasets, but these datasets need be relevant pertaining to the currency of the network intrusions. This paper focuses on a newly created dataset, *UWF-ZeekData22*, that analyzes data from Zeek’s Connection Logs collected using Security Onion 2 network security monitor and labelled using the MITRE ATT&CK framework TTPs. Due to the volume of data, Spark, in the big data framework, was used to run many of the well-known classifiers (naïve Bayes, random forest, decision tree, support vector classifier, gradient boosted trees, and logistic regression) to classify the reconnaissance and discovery tactics from this dataset. In addition to looking at the performance of these classifiers using Spark, scalability and response time were also analyzed.

## 1. Introduction and Background

Over the past decade, Internet of Things (IoT) traffic has increased exponentially. As more devices transfer data across networks in different sectors such as healthcare, agriculture, logistics, etc., network traffic is expected to increase exponentially, and it is predicted that by 2023, there will be at least 43 billion devices [1]. Hence, being able to monitor and recognize malicious activity and cyberattacks has become imperative. In this work, Zeek, an open-source network-monitoring tool that provides the raw network data needed to tackle today’s toughest networking challenges in the enterprise, cloud, and industrial computing environments, was used to collect data [2]. This one-of-a-kind new modern dataset, *UWF-ZeekData22* [3], was labeled using the MITRE Adversarial Tactics, Techniques, and Common Knowledge (ATT&CK) framework. The MITRE ATT&CK framework is a globally accessible knowledge base of adversary tactics and techniques used to accomplish specific objectives [4]. This work, specifically using Zeek’s Connection (Conn) Log files from the *UWF-ZeekData22* dataset [3], tries to identify connections that lead to the two adversary tactics, reconnaissance (TA0043) and discovery (TA0007). Users of the reconnaissance tactic gather information about vulnerabilities, which can be used for future attacks [5], and users of the discovery tactic try to better understand the internal network [6]. Zeek’s Conn log files track the protocols and associated information such as IP addresses, durations, transferred (two way) bytes, states, packets, and tunnel information. In short, Zeek’s Conn files provide all the data regarding the connection between two points [7].

Due to the volume of data involved, the Hadoop Distributed File System (HDFS) was used to store the data. HDFS is a scalable, fault-tolerant system that allows processing of big data by making it available across a cluster of computers [8]. An open-source framework for big data analytics that sits on top of the Hadoop framework, Apache Spark, was used for machine learning (ML). Spark’s machine learning algorithms, specifically decision tree (DT), gradient boosted trees (GBT), logistic regression (LR), naïve Bayes (NB), random forest (RF), and support vector machines (SVM), were used for binary classification in the big data framework. New binning methods were introduced to bin the raw network data, and feature reduction was performed using information gain. In addition to statistical metrics and execution timings obtained from the machine learning algorithms, an analysis was also performed to determine the ideal Spark parameter configurations for classifying a raw network dataset in the big data framework.

The uniqueness of this paper can be highlighted as follows: (i) this analysis is performed on a modern, one-of-a-kind, newly created Zeek MITRE ATT&CK framework labelled dataset, *UWF-ZeekData22*, which is available at datasets.uwf.edu (accessed on 20 August 2022) [3]; (ii) this is the first work to date that analyzes Zeek network connections that lead to the classification of the reconnaissance and discovery tactics as defined by the MITRE ATT&CK framework; and (iii) new binning methods are presented to bin a raw network dataset.

The rest of this paper is divided into the following sections. Section 2 presents the related works; Section 3 briefly explains how this new dataset was generated and the characteristics of this dataset; Section 4 explains the pre-processing in detail; Section 5 presents the machine learning algorithms and the Spark parameters used; Section 6 presents and discusses the results obtained; Section 7 presents the conclusion, and Section 8 presents the future works.

## 2. Related Works

Analysis of network data for the purpose of supporting an anomaly-based IDS has been a topic of interest for some time [9,10,11,12,13,14,15,16,17]. The first widely studied network dataset was the KDD99Cup dataset, analyzed in [10,12,15]. Reference [12] used a decision tree classifier to support anomaly-based intrusion detection in network data. They found that a particle swarm optimization algorithm can be used to prune trees and significantly improve model performance. Reference [15] showed that SVM, using principal component analysis for feature reduction, is effective for anomaly detection in a fog computing environment. Reference [10] used multiple learning algorithms (logistic regression, SVM, random forest, gradient boosting tree, and naïve Bayes) to test the performance differences that occur as the number of features are changed. They found that the accuracy of these learners is minimally affected by reducing the number of features, but training and testing time can be significantly reduced.

The next big dataset, NSL-KDD, is a smaller subset of the KDD99Cup dataset, with a significant number of duplicate records removed. It was analyzed by [10,13]. Reference [10] used both NSL-KDD and KDD99Cup and found that the larger, more redundant KDD99Cup dataset produced slower models as compared to the models developed using the smaller NSL-KDD dataset. Reference [13] combined different decision tree models and found that models perform best when combined with a sum-rule scheme.

The UNSW-NB15 dataset was created in 2015 by the University of New South Wales and features nine different attack types along with significantly more modern network traffic as compared to the KDD datasets. Reference [9] analyzed this dataset using the SVM, naïve Bayes, decision tree, and random forest classifiers on the Apache Spark framework and found that the random forest classifier performed the best in terms of both accuracy and execution time. Reference [18] analyzed this dataset on the Apache Spark framework using binary and multiclass random forest classifiers with principal component analysis and information gain as feature selection methods. They found that PCA made model training slower, and in some cases, accuracy was the same or worse than models trained without PCA. They also found that the binary classifier produces more accurate models than the multiclass classifier. Reference [19] analyzed UNSW-NB15 using both binary and multiclass random forest algorithms using information gain and principal component analysis as feature reduction methods and Apache Spark as the framework. They found that binary random forest outperforms multiclass random forest in terms of accuracy and that information gain outperforms principal component analysis in terms of both accuracy and speed.

Reference [17] analyzed CICIDS2017, and this work addresses issues with traffic diversity and volume, lack of metadata, outdated attack payloads, and anonymized packets found in other datasets. Reference [17] implemented k-nearest neighbor, random forest, decision tree, gradient boosting tree, multilayer perceptron, naïve Bayes, and quadratic discriminant analysis classifiers and reported their performance, stressing the importance of generating and analyzing updated IDS datasets to keep up with current network traffic/network attacks.

CSE-CIC-IDS2018, an update to CICIDS2017, followed a set of guidelines to create a systematic update to keep the IDS dataset up to date with current network trends. It was analyzed by [16], who applied gradient boosting tree, decision tree, random forest, naïve Bayes, and logistic regression classifiers to this dataset to determine the impact of feature selection and specifically the use of the Destination_Port feature. Reference [16] found that the Destination_Port feature was generally useful in terms of model accuracy and should be included when analyzing IDS data. This feature affected the performance of the machine learning algorithms. They found that feature selection algorithms produce models that perform as well as or better than models created without using feature selection algorithms while consuming fewer computing resources.

In addition to large, general-purpose IDS datasets, some studies have produced their own data tailored to their specific use cases. Reference [11] created and analyzed datasets of varying sizes labeled SYNDOS10K-SYNDOS2MIL, which were generated by making SYN DoS attacks on a network consisting of three PCs and nine IoT devices. They analyzed these datasets with logistic regression, decision tree, random forest, gradient boosting tree, and linear SVM and found that random forest performed best in terms of both accuracy and execution time on this type of data.

Other studies, such as [14], used data that are not made publicly available. They applied a multilayer SVM to their data to support a block lowest common ancestor algorithm to cluster network intrusion features. They found that this approach produces better clustering detection times when compared to similar methods.

There are very few works to date on the MITRE ATT&CK framework with respect to identifying attacks using machine learning. Reference [20] used two hidden layer feed-forward neural networks to impute missing values in a 2018/2019 ENISA dataset [21] based on ATT&CK descriptions of the intrusions. They found that this is a valid approach for filling out intrusion datasets with missing values. Reference [22] used hierarchical clustering on a small (270 records) intrusion dataset from MITRE to predict likely future attacks based on other recent attacks. They found that 75% of the ATT&CK techniques can be highly predicted based on earlier attacks by the same advanced persistent threat. Thus, though there are some works on the MITRE ATT&CK framework, there are no works that map a large, raw network dataset with attacks labeled according to the ATT&CK framework, which is then analyzed using machine learning.

## 3. Data

The Zeek Conn Log MITRE ATT&CK framework labeled dataset, *UWF-ZeekData22*, available at [3], generated using the Cyber Range at The University of West Florida (UWF), was used for this analysis. This dataset has 9,280,869 attack records and 9,281,599 benign records.

**Figure 1:**
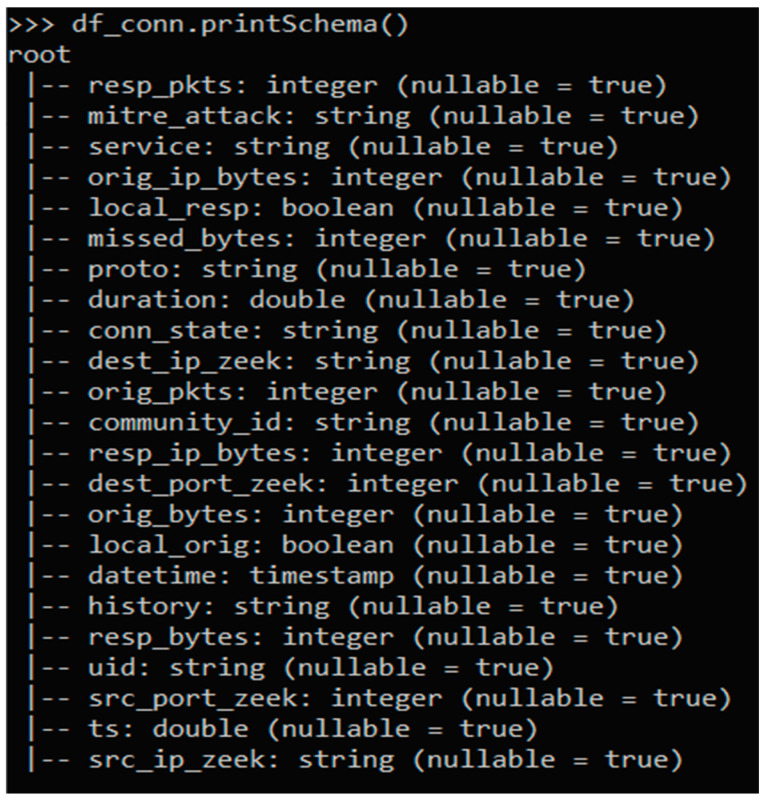
Schema of Conn Log File.

Zeek’s Conn log file tracks the protocols and associated information, such as IP addresses, durations, two-way bytes, states, packets, and tunnel information. In short, the Conn log files provide all the data regarding the connection between two points. The full list of the attributes (and attribute types) of the Conn log file is presented in Figure 1. The “mitre_attack” attribute was added to label the data as per the MITRE ATT&CK framework. A description of the attributes in the Zeek Conn Logs file is presented in Appendix A.

### 3.1. Data Breakdown by Tactic and Technique

Table 1 presents a breakdown of the data in the Zeek Conn logs by the MITRE ATT&CK Tactic. There were 9,278,722 instances of reconnaissance activity, 2086 instances of discovery activity, and very few instances of other adversary tactics in this dataset; hence, only reconnaissance and discovery were used for this analysis. Table 2 presents a breakdown of the Zeek Conn logs by the MITRE ATT&CK Technique, which is a more granular view of tactic (i.e., “T1046 Network Discovery Service” is a technique, which is a member of the discovery tactic).

### 3.2. The Reconnaissance Tactic

The reconnaissance tactic covers network attacks that are carried out with the goal of gathering information to plan future attacks [23]. This dataset has 9,278,722 reconnaissance records, all of which are T1595 active scanning attacks in the MITRE ATT&CK nomenclature [23], which could include scanning IP blocks, vulnerability scanning, or wordlist scanning.

### 3.3. The Discovery Tactic

The discovery tactic covers attacks that are meant to learn specifics of network infrastructure [5]. This dataset has 2086 discovery records, all of which are T1046 network service discovery attacks. In the T1046 technique, adversaries attempt to get a listing of services running on remote hosts and local network infrastructure. This is done through port scans or vulnerability scans [5].

## 4. Preprocessing

Preprocessing is broken down into two parts. The first part presents a way to preprocess raw network data using binning, and the second part uses information gain for feature selection.

### 4.1. Preprocessing Using Binning

The Zeek Conn logs contain continuous valued attributes, nominal attributes, IP addresses, and port numbers. For processing in Spark’s machine learning environment, preprocessing was required. The following sections cover how each type of column in the dataset (i.e., continuous value, nominal, IP number, and port) was preprocessed and binned.

#### 4.1.1. Binning Continuous Valued Columns

To establish the set of bin ranges for the continuous valued columns in this dataset, the trimmed mean and standard deviation was calculated for each column. To calculate the trimmed mean, first, null values as well as extreme outliers were removed. Several columns in this dataset (for example, duration) exhibit skewed data distributions, often forming long tails skewing to the right. To address the skewness caused by lengthy and low-lying tails, a 10% trim on the data was used to generate the bins. This resulted in 80% of these data being used for mean and standard deviation calculations, with 10% of the lowest-ranking and highest-ranking values being removed. The binning can be outlined with the following edges:

edge0 = float(‘−inf’)
edge1 = mean_val − stddev_val × 2
edge2 = mean_val − stddev_val
edge3 = mean_val
edge4 = mean_val + stddev_val
edge5 = mean_val + stddev_val × 2
edge6 = float(‘inf’)
edges = [edge0, edge1, edge2, edge3, edge4, edge5, edge6]

**Figure 2:**
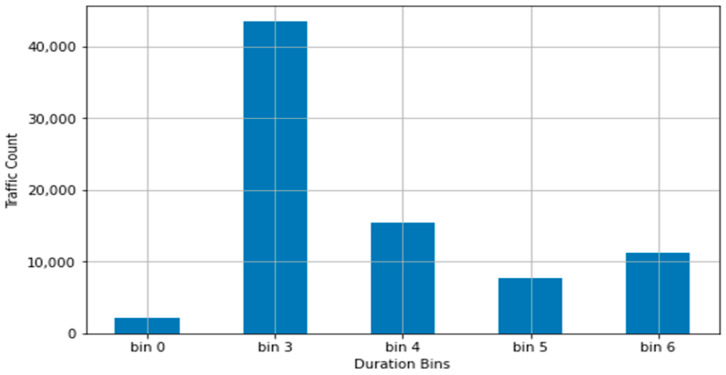
Initial value distribution for duration across bins.

The bin ranges were used in conjunction with the PySpark ML Bucketizer function to convert the respective columns into a limited range of integer values. The resulting bins and distribution for the duration column, using the above binning outline, are presented in Table 3 and Figure 2.

**Figure 3:**
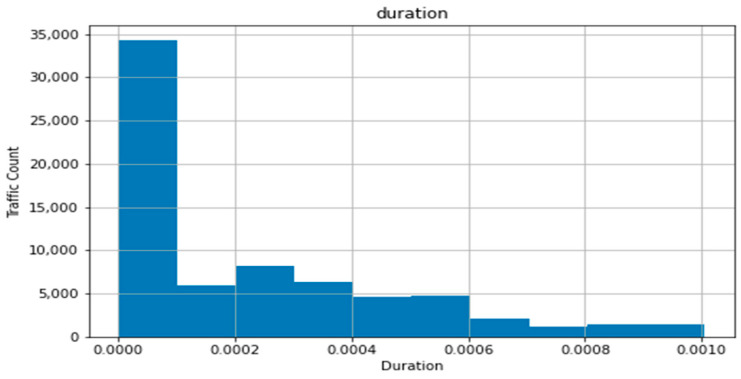
Normal distribution of duration with improved standard deviation.

The resulting distribution for duration, with bin1 and bin2 ending in a negative value for their high end and having a 0 count of values, was caused by the slant of the dataset, resulting in two standard deviations left of the dataset’s mean pushing over the 0 value. This difference with a normal distribution, where the application of the standard deviation is ideal, can be seen in Figure 3, generated using the maximum value of the trimmed dataset, which clearly shows a heavily leftwards distribution.

##### Using the Moving-Mean to Bin Continuous Valued Columns

To maintain the desired number of bins in spite of the skewness of the data and to avoid redundant bin ranges, a moving-mean logic was inserted during the establishment of the edges (where the minimum value of an attribute never drops below 0):

if min_val ≥ 0:
 while mean_val—2 × stddev_val < 0:
  mean_val += stddev_val

This moving-mean logic would insert itself in the binning method such that the bins established through a set number of standard deviations within the mean value of the trimmed dataset would shift rightwards with the moving-mean value until the first two bins no longer encompassed a redundant range of data. Table 4 presents the distribution with this process performed on the duration column.

**Figure 4:**
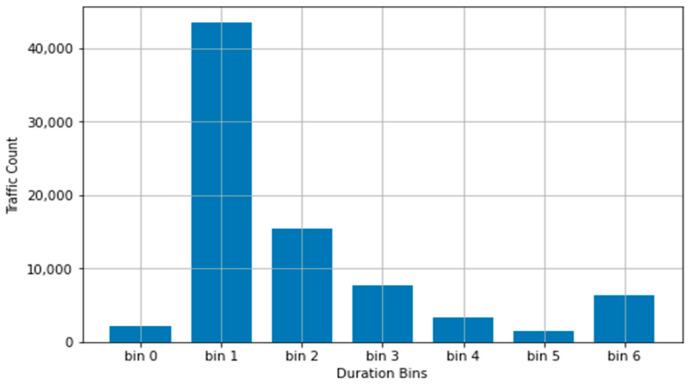
Value distribution for duration across bins after moving-mean logic.

As can be seen in Figure 4, the moving-mean generated two additional bins, taking away from the previously established bin6. Previously representing a bin range of duration values more than three standard deviations away from the true mean of the trimmed dataset, this would now (after the emergence of two additional bins) encompass values more than five standard deviations from the true mean.

The parity of box6 in both sets of results, as well as the obvious positive skewness leftward, is contradictory to the nature of normal distributions. This, however, must be taken with the general level of abstraction inherent in any binning methodology.

Table 5 shows the bin counts for each attribute when this binning method is applied to the continuous attributes in the Zeek Conn file. The moving-mean bins were also calculated. An additional bin was generated for null values with a bin designation of 0.

#### 4.1.2. Binning Nominal Valued Columns

Nominal values within this dataset are columns containing non-numeric data. They may contain names of things, categories, states, or sequences. They often contain non-numeric characters although that is not always the case. One of the difficulties in their consideration in algorithms is that such data can contain many unique values, whose naming conventions do not necessarily indicate an intrinsic value difference. For this analysis, non-numeric values were converted into numbers. Spark provides a convenient means of performing such conversions through the StringIndexer method from MLib [6], which is Apache Spark’s scalable machine learning library. This method was set up to keep all invalid or empty values, converting those as well to separate integer values for binning purposes. This method was applied to the columns presented in Table 6, with the resulting bin counts.

Though the IP addresses and port numbers would also fall under this category, they were handled as explained in the next couple sections.

#### 4.1.3. Binning IP Address Columns

For binning IP addresses of traffic source and destination, the commonly recognized network classifications of A, B, C, D, and E were used, each of which pertain to specific ranges of the first octet in the IP address [24]. These different octet ranges correlate to different default subnet masks, with ascending classifications reserving more bits for the network address and fewer for the host address. Class A is best-suited for serving incredibly large networks, while Class C would normally be assigned to very small networks. Classes D and E are normally relegated to experimental use cases, such as for multicasting, research, and development [24].

Below are the established octet ranges (inclusive) for each classification [24]:

- Class A: First octet value 0–126;
- Class B: First octet value 128–191;
- Class C: First octet value 192–223;
- Class D: First octet value 224–239;
- Class E: First octet value 240–254.

Using these ranges, the data in these attributes were binned into seven categories, as shown in Table 7. The “Other” classification was used to capture values that may exceed these boundaries, and a category was used for null or non-applicable values. An example of this classification method was applied to the dest_ip column of the Zeek Conn data, as shown in Table 7. This column represents the IP address of a packet destination on the network, and it totaled 312 unique addresses within the scope of the sample dataset.

Based on Table 5, Classes A and B IP addresses were the most plentiful in this data. This is to be expected given that Classes A, B, and C are considered the most commonly occurring IP types in most network traffic [24]. The remaining bins represent a small fraction of the total dataset.

Applied to both of the IP columns in the Zeek Conn data, Table 8 shows the changes in unique values before and after binning.

#### 4.1.4. Binning Port Numbers

Port numbers can range widely in value, with the Internet Assigned Numbers Authority (IANA) administering ports 0 through 65,535 [25]. This spectrum can be divided into three ranges covering well-known ports, registered ports, and dynamic/private ports. Well-known ports range from 0 to 1023 and are protected, with operating systems restricting access to these ports to only processes with appropriate privileges. Registered ports range from 1024 to 49,151, and their use should only be with IANA registration. All other ports up to 65,353 can be used more freely for all manner of purposes.

Based on this classification system, Table 9 shows the bin ranges that were generated to represent the three port ranges, with a bin value of 0 representing null values. With the bin ranges applied in Table 9, the results for dest_port and src_port are as shown in Table 10.

The difference between the two port types (src_port and dest_port) within this dataset is also more clearly defined, with most of the source network traffic originating from registered and dynamic ports and the destination ports largely made up of the well-known ports, as can be observed in Table 11.

### 4.2. Information Gain

After binning, information gain was used to assess the relevance of the 18 features. Information gain is the difference between a class’s entropy and the entropy of the class and a selected feature split, with entropy measuring the extent of randomness in the dataset [27]. It is an assessment of the usefulness of a feature in classification.

The following calculations [28] were performed on each feature to produce information gain values for ranking purposes:

(1) $$Gain\left(A\right)=Info\left(D\right)-Info_{A}\left(D\right)$$

where

(2) $$Info\left(D\right)=-\overset{m}{\underset{i=1}{\sum }}p_{i}log_{2}\left(pi\right)$$

(3) $$Info_{A}\left(D\right)=\overset{V}{\underset{j=1}{\sum }}\frac{\left|D_{j}\right|}{\left|D\right|}\times Info\left(D_{j}\right)$$

Given that

- *Info(D)* is the average amount of information needed to identify the class level of a tuple in *D*;
- *Info_A_(D)* is the expected information required to classify a tuple from *D* based on partitioning from *A*;
- *p_i_* is the nonzero probability that an arbitrary tuple belongs to a class;
- *|D_j_|/|D|* is the weight of the partition.

The information gain values for the features (attributes) in the Zeek Conn logs are presented in Table 12.

The higher the attribute on the list, the more relevant it is in the classification process. Based on these results, the top 6, top 9, top 12, and top 18 attributes were used to run the machine learning algorithms.

## 5. Experimentation

**Figure 5:**
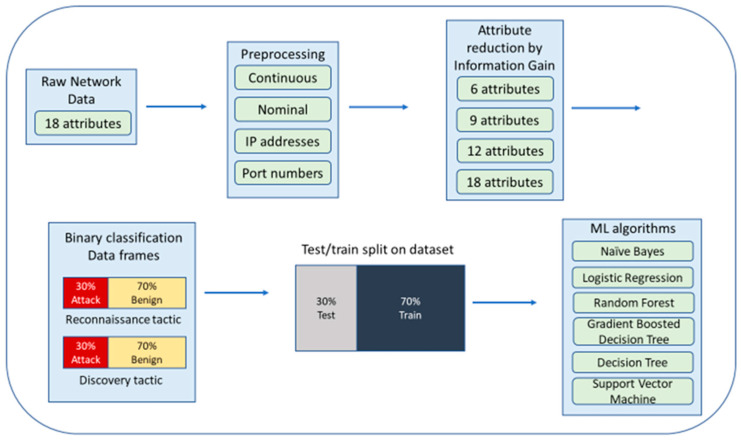
Experimental flow.

Figure 5 presents the experimentation process. First, after binning the raw network data, information gain was calculated. Then, the two tactics, reconnaissance and discovery, were isolated and segregated into separate dataframes for binary classification. The ratio of attack to benign data was maintained at 30%:70% in each dataframe. The dataset was split into 70:30 for training and testing the machine learning classifiers. For each machine learning algorithm, four sets of attributes were tested: 6, 9, 12, and 18.

### 5.1. Computing Environment

The University of West Florida’s Hadoop cluster was used. This cluster consists of six Dell PowerEdge R730XD servers and three Dell PowerEdge R730 servers, each of which has dual Intel Xeon E5-2650v3 CPUs with 10 cores and 20 threads per CPU (40 cores) and 128 GB of DDR4 RDIMM RAM. The cluster has six worker nodes that execute the machine learning algorithms. Spark 3.2.1 and Hadoop 3.3.1 were used for the environment. Spark provides a rich set of APIs that were used to implement and run the various machine learning algorithms [29].

### 5.2. Machine Learning Algorithms Used

Six different classifiers were compared: logistic regression, naïve Bayes, random forest, gradient boosted tree, decision tree, and support vector machines. Spark’s Machine learning libraries were used to run the various classifiers, and the *BinaryClassificationMetrics* was used to generate various statistical results such as accuracy, precision, recall, F-measure, area under the curve, and false-positive rate.

#### 5.2.1. Logistic Regression

Logistic regression (LR) applies a linear discriminant rule and is a widely used machine learning algorithm used for classification of data [11]. Logistic regression considers each feature by associating a specific weight to it to generate a probability of being classified to a specific class. Larger weights represent more variation in a feature and have a larger impact on the algorithm. Binary responses can be predicted by making use of binary logistic regression. Spark 3.2.1′s *pyspark.ml.classification.LogisticRegression* implementation was used in this study, and the parameters are specified in Table 13.

#### 5.2.2. Naïve Bayes

Naïve Bayes (NB) is a popular machine learning algorithm that uses the Bayes rule of classification and assumes independence of features [10]. The model is easy to build and intuitive [10], and studies have shown that it works with high accuracy for smaller datasets. Spark 3.2.1′s *pyspark.ml.classification.NaiveBayes* implementation was used in this study, and all parameters used are default values, as shown in Table 13.

#### 5.2.3. Random Forest

Random forest (RF) is an ensemble method; multiple decision trees are constructed, with each tree being built from a limited number of the available features. The classification is performed by polling the collection of trees, and a majority vote is used for this. Spark 3.2.1′s *pyspark.ml.classification.RandomForestClassifier* was used in this study, with the default settings shown in Table 13.

#### 5.2.4. Gradient Boosted Tree

Gradient boosted tree (GBT) is another class of ensemble methods with many popular implementations. Gradient boosted tree is based on constructing multiple decision trees in sequence, with each decision tree being made in such a way as to minimize the classification error rate of the previous tree(s). Spark 3.2.1′s *pyspark.ml.classification.GBTClassifier* implementation was used in this study (this is an implementation of stochastic gradient boosting [30]), and the default parameters were used, as shown in Table 13.

#### 5.2.5. Decision Tree

A decision tree (DT) is a set of nodes that partitions the data and returns a binary decision (yes or no) given the node’s condition. Based on the decision made, the corresponding child node is given the input vector. This continues until a leaf node is reached, and the final decision is made. The Spark implementation supports decision trees for binary and multi classification and for regression. The creation of the Spark’s decision tree is based on information gain and works through a greedy algorithm that performs recursive binary partitioning.

Spark 3.2.1′s *pyspark.ml.classification.DecisionTreeClassifier* implementation was used in this study. Most default parameters were used, as presented in Table 13, except for maxBins and MaxDepth. Due to the binning methods used, none of the features used in the training and test phases contained more than 60 unique values. A max bin value was set for 100, just in case any of the nominal attributes had significantly more in larger datasets. To further account for this possibility, the potential depth of the resulting classifier tree was increased from 5 to 100, at the expense of performance, to ensure a large degree of granularity.

#### 5.2.6. Support Vector Machines

Support vector machines (SVM) is a supervised machine learning algorithm used for regression and classification. The goal of the SVM algorithm is to find an optimal hyperplane (also known as a classification vector) in a multi-dimensional space, which allows for the classification of data points relative to their position to the hyperplane. Linear SVM in Spark machine learning supports binary classification. Internally, it optimizes the hinge loss using the OWLQN optimizer. Spark 3.2.1′s *pyspark.ml.classification.LinearSVM* implementation was used in this study, and the default settings were used, as shown in Table 13.

## 6. Results

The results are presented in two parts. The first part presents the results for testing the various Spark parameters in the big data environment, and the second part presents the results of the machine learning classifiers run with the best-performing Spark parameters (determined in the first part).

### 6.1. Testing Spark’s Configuration Parameters

Spark’s configuration parameters, as shown in Table 14, were declared during the creation of the Spark session and dictated the available resources that the algorithms were allocated.

#### 6.1.1. Performance Results with Various Executor Parameters

The decision tree classifier, with 18 attributes, was used to analyze the executor parameters. Table 15 shows the effect of executor count, executor core count, total executor cores, executor memory, and total executor memory on binning time and training time in seconds. Table 16 shows the total time with additional parameters such as driver memory, driver cores, and shuffle partitions and how they affect total time.

For the rest of the figures in this section, the binning time, training time, and testing time were combined into a single value called “process time”.

**Figure 6:**
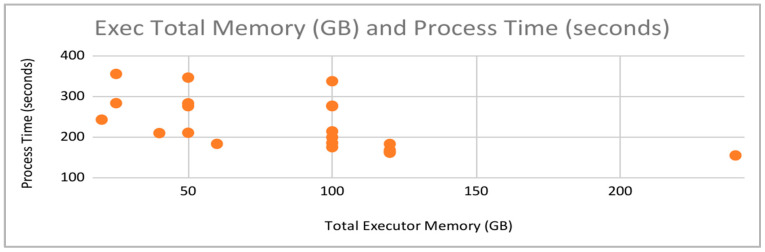
Process Times vs. Memory per Executor.

Figure 6 presents process times versus the memory per executor. Executor memory is the amount of memory (in GBs) assigned to each executor for use with their assigned cores. From Figure 6, there does not appear to be a strong correlation between the total executor memory and processing time. While a necessary component for utilizing the executors, actual performance improvements could be more accurately attributed to other parameter options. Fine tuning of other parameters could involve reducing the amount of unnecessary memory assigned to executors, hence increasing available resources on the server.

**Figure 7:**
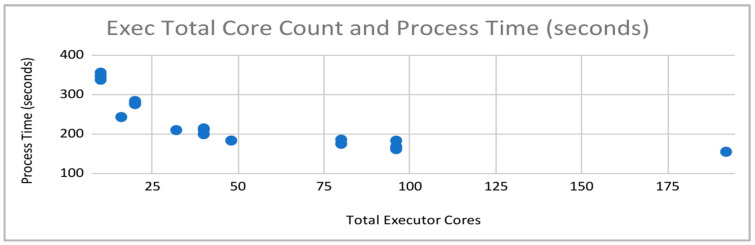
Process Times vs. Total Number of Executor Cores.

There was a more noticeable effect on performance, albeit with diminishing returns, with the number of executors instantiated and the number of cores assigned to each of them. Multiplying these two values, we arrive at total executor cores. As can be seen in Figure 7, increasing the number of cores available from 10 to 50 had a significant impact on performance, with the aforementioned diminishing returns becoming readily apparent beyond that count and especially after a total core count of 100.

**Figure 8:**
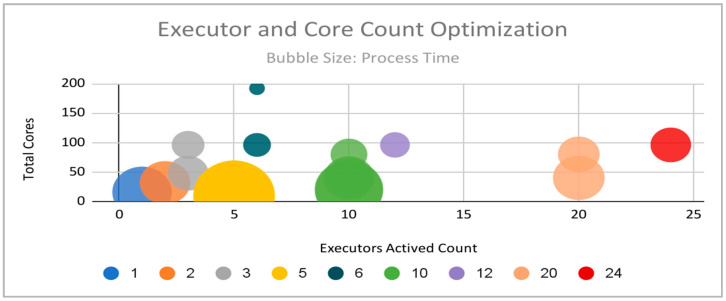
Processing times by number of executors and total number of cores across all executors.

Figure 8 sheds light on the optimal number of executor instances given the total number of cores assigned to the process. The smaller the circle, the less processing time (that is, binning time + training time + testing time) it took to generate the results. The total number of executor cores had a high impact on improving the processing time. This observation is born out here as well, with higher values of total cores showing smaller circles.

An interesting observation can be made along the horizontal line representing 96 total cores (approx. 100). Along this axis are test results for executor counts at 3, 6, 12, and 24. All other parameters being equal, the best results at this core count range were shown with six executor instances. While the best performance in Figure 8 (based on the smallest circle) is given by the lone results at a total core count of 196 (approx. 200), the 96 total core count option with six executors was regarded as the best and hence used to test the overall performance of the machine learning algorithms.

**Figure 9:**
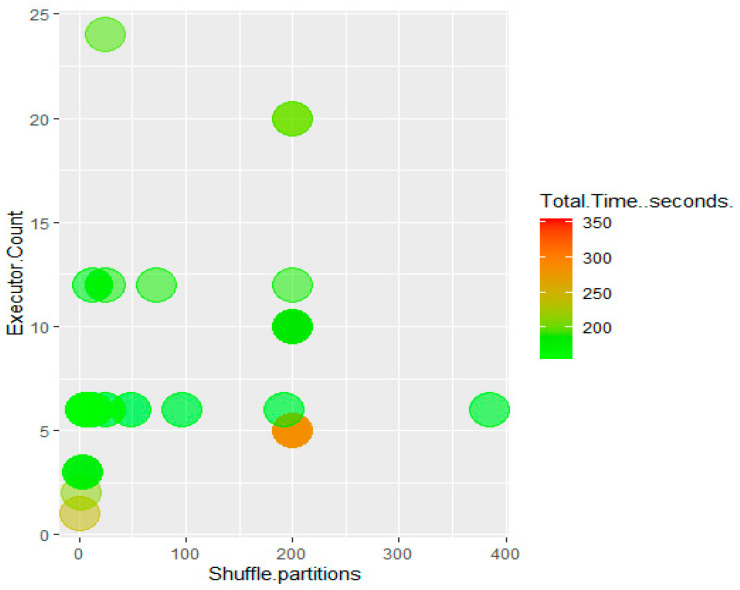
Processing times as a function of executor count and shuffle partitions.

**Figure 10:**
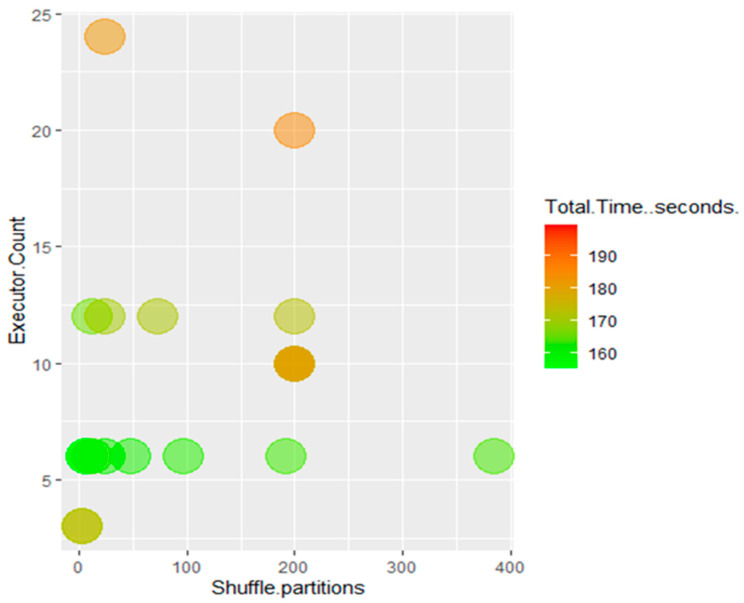
Processing times as a function of executor count and shuffle partitions, Time ≤ 200 s.

The shuffle partitions parameter was also tested. This is displayed with executor count and performance in Figure 9 and Figure 10. Trends at both 6 and 12 executors show that using fewer shuffle partitions improves performance, with the fastest performance occurring when the number of shuffle partitions is the same as the total number of executors. Values for the shuffle partitions attribute lower than the total number of executors allocated were not tested, as this would leave executors beyond the number of shuffle partitions idle.

We elected to use the parameters detailed in Test ID 19 from Table 16. This is not the fastest performance, but it was less than 3% slower than our fastest settings while using half of the total resources. Table 17 and Table 18 show these spark configuration parameter settings.

#### 6.1.2. Effect on Training Time

**Figure 11:**
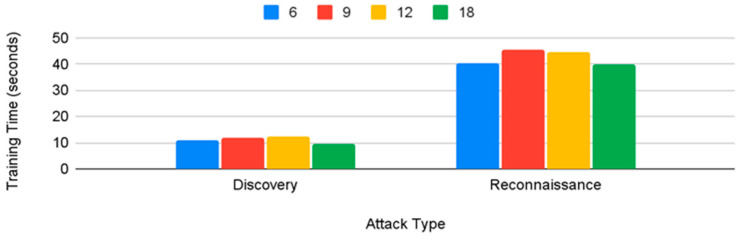
Number of attributes vs. training times for all algorithms.

As shown in Figure 11, the number of attributes used does not seem to strongly correlate to model training time. The differences in training time between discovery and reconnaissance can be attributed to the size difference between the training sets; the discovery training set contains around 6000 records, while the reconnaissance training set contains around 20,000,000 records.

### 6.2. Machine Learning Performance

This section presents the results of binary classification performed on the Zeek Conn Log data using the six machine learning algorithms (decision tree, gradient boosting tree, logistic regression, naïve Bayes, random forest, and support vector machine) using the best parameters determined from the previous section (as shown in Table 17 and Table 18).

#### 6.2.1. Evaluation Metrics

Results were collected for accuracy, precision, recall, false-positive rate (FPR), F-measure, area under operating characteristics curve (AUROC), and training/testing times in seconds. Next, these metrics are defined.

Accuracy: Accuracy is the number of correct classifications (i.e., true positives and negatives) divided by the total number of classifications [32].

Accuracy = [True Positives + True Negatives]/ [True Positives + False Positives + True Negatives + False Negatives](4)

Precision: Precision, also known as confidence, is the proportion of predicted positive cases that are correctly labeled as positive [33]. Precision by label considers only one class and measures the number of times a specific label was predicted correctly, normalized by the number of times that label appears in the output.

Precision = Positive Predictive Value = [True Positives]/[True Positives + False Positives] (5)

Recall: Intuitively, recall is the ability of the classifier to find all the positive samples, i.e., the true-positive rate. Recall is also known as sensitivity and is the proportion of real positive (RP) cases that are correctly predicted as positive (TP) [33].

All Real Positives = [True Positives + False Negatives](6)

All Real Negatives = [True Negatives + False Positives](7)

Recall = True Positive Rate = [True Positives]/[All Real Positives](8)

False-positive rate (FPR): This is the proportion of negative labels that are predicted to be positive.

False Positive Rate (FPR) = [False Positives]/[All Real Negatives](9)

F-Measure: The F-measure is defined as the harmonic mean of a prediction’s precision and recall metrics. It is another overall measure of the test’s accuracy [34].

F-Measure = 2 × [Precision × Recall]/[Precision + Recall](10)

AUROC: A receiver operating characteristics (ROC) graph is a technique for visualizing, organizing, and selecting classifiers based on their performance. ROC graphs depict the relative tradeoffs between the true and false positives. They are two-dimensional graphs upon which classifiers are plotted, with the *y*-axis being the true-positive rate and the *x*-axis the false-positive rate (FPR) [35].

The area under ROC (AUROC) is the percentage of the area under the resulting line relative to the entire area of the graph [36].

Recall, sensitivity, and TPR connate the same measure.

Training and testing time: The time it took for an algorithm to complete its training and testing processes (in seconds) is recorded in these two columns, respectively. All other factors being equal, an algorithm is considered to have performed better than another if it completed its calculations more quickly.

#### 6.2.2. Machine Learning Classifier Results

Binary classification was performed using each of the six machine learning classifiers for 6, 9, 12, and 18 attributes.

##### Machine Learning Classifier Results for Reconnaissance

Machine learning classifier results for the reconnaissance tactic for 6, 9, 12, and 18 attributes are presented in Table 19.

From Table 19, for the reconnaissance tactic, it can be noted that, in terms of accuracy, decision tree, gradient boosting tree, and random forest had the highest averages for all sets of attributes. Naïve Bayes had the lowest accuracy, and logistic regression was only slightly higher than naïve Bayes. In terms of recall, gradient boosting tree and random forest had the highest recall, followed by decision tree. Naïve Bayes, support vector machine, and logistic regression had lower recall rates. In terms of false-positive rates, it can be noted that decision tree had the lowest false-positive rates, and support vector machine and gradient boosting tree had the second lowest false-positive rates. Naïve Bayes had the highest false-positive rates. In terms of training time, random forest had the lowest training time, followed by decision tree, for all attribute combinations. Gradient boosting tree had the highest training times.

##### Machine Learning Classifier Results for Discovery

Machine learning classifier results for the discovery tactic for 6, 9, 12, and 18 attributes are presented in Table 20.

From Table 20, for the discovery tactic, in terms of accuracy, it can be noted that, decision tree, gradient boosting tree, and random forest had a higher accuracy for all sets of attributes. In terms of recall, gradient boosting tree, naïve Bayes, and logistic regression had higher recall for all sets of attributes, and decision tree was close behind. Support vector machine performed poorly in terms of recall. In terms of the false-positive rates, decision tree, gradient boosting tree, and random forest all had lower false-positive rates for all combination of attributes although decision tree appeared to perform the best.

#### 6.2.3. Overall Results for Reconnaissance and Discovery

**Figure 12:**
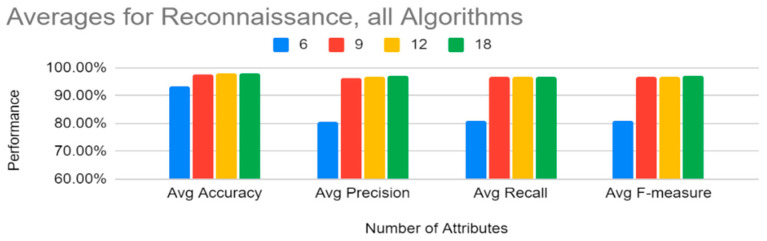
Reconnaissance: Averages for All Algorithms by Number of Features.

**Figure 13:**
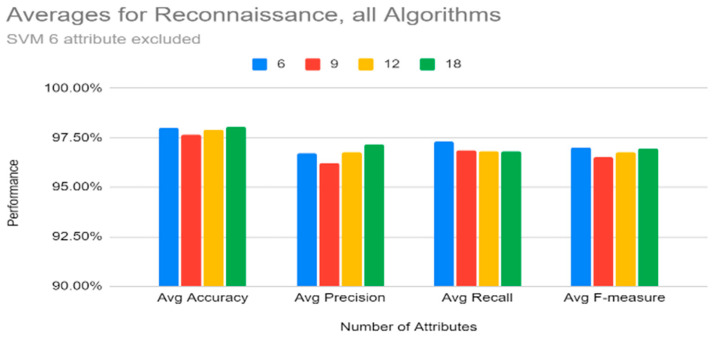
Reconnaissance: Averages for All Algorithms by Number of Features (SVM’s six attributes excluded).

Figure 12 shows that, for reconnaissance, on average, 6 attributes performed slightly lower than 9, 12, and 18 attributes. However, a close look at Table 19 shows that this is because the SVM results for six attributes are not consistent with the rest of the results; hence, Figure 13 shows averages for reconnaissance without SVM (note that SVM was just removed from the six attribute list). Without SVM’s six attributes, all the other results are higher for six attributes.

From Figure 11, we can also note that the training time was lower, on the average, for six attributes. Therefore, for reconnaissance, the top six attributes (history, protocol, service, orig_bytes, dest_ip, and orig_pkts) would be enough to classify the reconnaissance tactic for any of the classifiers except SVM.

**Figure 14:**
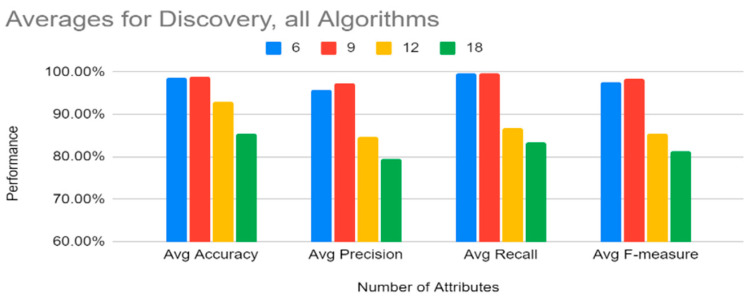
Discovery: Averages for All Algorithms by Number of Features Used.

Figure 14 shows that, for discovery, on average, 18 attributes performed the lowest, and though 6 and 9 attributes performed closely, 9 attributes had slightly higher averages. However, since the top six attributes (history, protocol, service, orig_bytes, dest_ip, and orig_pkts) had results so close to the top nine attributes, the case could be made to recommend six attributes given that, in terms of training time as shown in Figure 11, six attributes performed better than nine attributes.

## 7. Conclusions

The objective of this paper was to see if the reconnaissance and discovery tactics, labelled using the MITRE ATT&CK framework, could be identified from the Zeek Conn logs using a newly created dataset, *UWF-ZeekData22* [3]. In addition to looking at the performance of these classifiers using Spark, scalability and response time were also analyzed.

In terms of optimizing classifier performance on the Spark cluster, we found that more total cores provided to the Spark application make machine learning algorithms run faster but with diminishing returns. It can be noted that classifiers run fastest when the number of shuffle partitions is the same as the total number of executors. There was no significant correlation between runtimes and the total amount of memory allocated (though allocating too little memory can cause executors to crash).

Machine learning results indicate that the tree-based methods (decision tree, gradient boosting tree, and random forest) performed better on most metrics than the other three algorithms in classifying this dataset for both the reconnaissance and discovery tactics. These three algorithms all showed 99% + accuracy for both attack tactics, with similarly higher scores in precision, recall, f-measure, and AUROC. Of the tree-based methods, gradient boosting tree and random forest performed a little better than decision tree in terms of recall for both the tactics, but in terms of the false-positive rate, decision tree had the lowest false-positive rates for both reconnaissance and discovery (in fact, it was at 0% for discovery). Gradient boosting tree and random forest also performed well in terms of false-positive rate for discovery, but for reconnaissance, gradient boosting tree performed a little better than random forest. Based on these results, it should also be mentioned that the binning methods used in this study were also effective in the classification process.

With respect to the training times of the tree-based classifiers, random forest performed the best for the reconnaissance tactic, followed by decision tree, and for the discovery tactic, decision tree performed the best.

With respect to the number of attributes to be used, the top six attributes from information gain (history, protocol, service, orig_bytes, dest_ip, and orig_pkts) can be considered enough to provide the best classification results for both the reconnaissance and discovery tactics for all the machine learning classifiers (except SVM with respect to reconnaissance), and the training time of the top six attributes was also lower.

## 8. Future Work

This work analyzes Zeek network connections and classifies them based on the reconnaissance and discovery tactics as defined by the MITRE ATT&CK framework. Although we only labelled the attacks based on tactics, we will extend this work to reflect the attack chain and techniques with corresponding IDs from the ATT&CK framework. Using the ATT&CK framework tactics, techniques, and procedures, we intend to analyze more attack traffic and label them with appropriate attack techniques. This will eventually provide a rich dataset for the research community to conduct more in-depth and relevant research on attack classification and pattern recognition using the MITRE ATT&CK mapping.

## Figures and Tables

**Table 1:** Conn log breakdown by MITRE ATT&CK tactic.

Label_Tactic	Count
Discovery	2086
Lateral movement	4
Privilege escalation	13
Reconnaissance	9,278,722
Persistence	1
Initial access	1
Exfiltration	7
Defense evasion	1
Resource development	3
Credential access	31
Benign data	9,281,599

**Table 2:** Conn log breakdown by MITRE ATT&CK technique.

MITRE ATT&CK Technique	Count
T1585	9,278,722
T1078	1
T1048	7
T1068	12
T1046	2086
T1588	3
T1021	4
T1552	31

**Table 3:** Distribution for Duration.

Bin Range	Bin Value	Count
Bin1	1	-
Bin2	2	-
Bin3	3	43,548
Bin4	4	15,458
Bin5	5	7656
Bin6	6	11,208

**Table 4:** Distribution for duration with moving-mean logic.

Bin Range	Bin Value	Count
Bin1	1	43,548
Bin2	2	15,458
Bin3	3	7656
Bin4	4	3382
Bin5	5	1506
Bin6	6	6320

**Table 5:** Number of bins for continuous attributes before and after moving-mean.

	Bins Used (Nulls Included)	Moving-Mean Bins (Nulls Included)
duration	5	7
orig_bytes	5	7
orig_pkts	5	5
orig_ip_bytes	6	6
resp_bytes	2	2
resp_pkts	4	6
resp_ip_bytes	4	6
missed_bytes	1	1

**Table 6:** Number of bins for integer attributes in Zeek Conn data.

	Counts of Integer Bins
proto	3
conn_state	10
local_orig	2
local_resp	2
history	59
service	10

**Table 7:** Binning illustrated for dest_ip attribute in dataset.

Classification	Dest_Ip Bin	Count
Class A	1	33,039
Class B	2	46,063
Class C	3	363
Class D	4	57
Class E	5	0
Other	6	5
Null/Non-applicable	0	569

**Table 8:** Number of unique values before and after binning for dest_ip and src_ip attributes.

	Unique Values before Binning	Unique Values after Binning
dest_ip_zeek	312	6
src_ip_zeek	39	3

**Table 9:** Binning shown for port number attributes.

Classification	Range	Bin Value
Well-known ports	0–1023	1
Registered ports	1024–49,151	2
Dynamic/Private ports	49,152–65,535	3
Null values	Null	0

**Table 10:** Number of unique values before and after binning dest_port and src_port.

Bin Values	Classification	Src_Port Count	Dest_Port Count
0	Null	0	0
1	Well-known ports	980	49,003
2	Registered ports	45,417	28,957
3	Dynamic/Private ports	33,699	2136
4	Other	0	0

**Table 11:** Differences between bin values for src_port vs. dest_port [26].

Bin Values	Classification	Src_Port Count	Dest_Port Count
0	Null	0	0
1	Well-known ports	980	49,003
2	Registered ports	45,417	28,957
3	Dynamic/Private ports	33,699	2136
4	Other	0	0

**Table 12:** Information gain for features in Zeek Conn dataset.

Attribute No.	Attribute	Info_Gain
1	history	0.827
2	protocol	0.77
3	service	0.726
4	orig_bytes	0.724
5	dest_ip	0.674
6	orig_pkts	0.655
7	orig_ip_bytes	0.572
8	local_resp	0.524
9	dest_port	0.486
10	duration	0.386
11	conn_state	0.166
12	resp_pkts	0.085
13	resp_ip_bytes	0.065
14	src_port	0.008
15	resp_bytes	0.008
16	src_ip	0.007
17	local_orig	0.002
18	missed_bytes	0

**Table 13:** Parameters specified for machine learning algorithm implemented in PySpark.

Machine Learning Algorithm	Parameters Used
**Logistic Regression**	featuresCol: str = **‘features’**, labelCol: str = **‘label’**, predictionCol: str = **‘prediction’**, maxIter: int = **10**, regParam: float = **0.3**, elasticNetParam: float = **0.8**, tol: float = **1 ×10^−6^**, fitIntercept: bool = **True**, threshold: float = **0.5**, thresholds: Optional[List[float]] = **None**, probabilityCol: str = **‘probability’**, rawPredictionCol: str = **‘rawPrediction’**, standardization: bool = **True**, weightCol: Optional[str] = **None**, aggregationDepth: int = **2**, family: str = **‘auto’**, lowerBoundsOnCoefficients: Optional[pyspark.ml.linalg.Matrix] = **None**, upperBoundsOnCoefficients: Optional[pyspark.ml.linalg.Matrix] = **None**, lowerBoundsOnIntercepts: Optional[pyspark.ml.linalg.Vector] = **None**, upperBoundsOnIntercepts: Optional[pyspark.ml.linalg.Vector] = **None**, maxBlockSizeInMB: float = **0.0**
**Naïve Bayes**	featuresCol: str = **‘features’**, labelCol: str = **‘label’**, predictionCol: str = **‘prediction’**, probabilityCol: str = **‘probability’**, rawPredictionCol: str = **‘rawPrediction’**, smoothing: float = **1.0**, modelType: str = **‘multinomial’**, thresholds: Optional[List[float]] = **None**, weightCol: Optional[str] = **None**
**Random Forest**	featuresCol: str = **‘features’**, labelCol: str = **‘label’**, predictionCol: str = **‘prediction’**, probabilityCol: str = **‘probability’**, rawPredictionCol: str = **‘rawPrediction’**, maxDepth: int = **5**, maxBins: int = **32**, minInstancesPerNode: int = **1**, minInfoGain: float = **0.0**, maxMemoryInMB: int = **256**, cacheNodeIds: bool = **False**, checkpointInterval: int = **10**, impurity: str = **‘gini’**, numTrees: int = **20**, featureSubsetStrategy: str = **‘auto’**, seed: Optional[int] = **None**, subsamplingRate: float = **1.0**, leafCol: str = **‘‘**, minWeightFractionPerNode: float = **0.0**, weightCol: Optional[str] = **None**, bootstrap: Optional[bool] = **True**
**Gradient Boosted Decision Tree**	featuresCol: str = **‘features’**, labelCol: str = **‘label’**, predictionCol: str = **‘prediction’**, maxDepth: int = **5**, maxBins: int = **32**, minInstancesPerNode: int = **1**, minInfoGain: float = **0.0**, maxMemoryInMB: int = **256**, cacheNodeIds: bool = **False**, checkpointInterval: int = **10**, lossType: str = **‘logistic’**, maxIter: int = **20**, stepSize: float = **0.1**, seed: Optional[int] = **None**, subsamplingRate: float = **1.0**, impurity: str = **‘variance’**, featureSubsetStrategy: str = **‘all’**, validationTol: float = **0.01**, validationIndicatorCol: Optional[str] = **None**, leafCol: str = **‘‘**, minWeightFractionPerNode: float = **0.0**, weightCol: Optional[str] = **None**
**Decision Tree**	featuresCol: str = **‘features’**, labelCol: str = **“label_bin”**, predictionCol: str = **‘prediction’**, probabilityCol: str = **‘probability’**, rawPredictionCol: str = **‘rawPrediction’**, maxDepth: int = **30**, maxBins: int = **100**, minInstancesPerNode: int = **1**, minInfoGain: float = **0.0**, maxMemoryInMB: int = **256**, cacheNodeIds: bool = **False**, checkpointInterval: int = **10**, impurity: str = **‘gini’**, seed: Optional[int] = **None**, weightCol: Optional[str] = **None**, leafCol: str = **‘‘**, minWeightFractionPerNode: float = **0.0**
**SVM**	featuresCol: str = **‘features’**, labelCol: str = **‘label_bin’**, predictionCol: str = **‘prediction’**, maxIter: int = **100**, regParam: float = **0.0**, tol: float = **1 ×10^−6^**, rawPredictionCol: str = **‘rawPrediction’**, fitIntercept: bool = **True**, standardization: bool = **True**, threshold: float = **0.0**, weightCol: Optional[str] = **None**, aggregationDepth: int = **2**, maxBlockSizeInMB: float = **0.0**

**Table 14:** Spark configuration parameters [31].

Configuration	Spark Property	Description
Driver Cores	spark.driver.cores	Number of cores used for the driver process in cluster mode.
Driver Memory	spark.driver.memory	Amount of memory used for the driver process, i.e., where Spark Context is initialized.
Executor Cores	spark.executor.cores	Number of cores used on each executor.
Executor Memory	spark.executor.memory	Amount of memory used per executor process.
Executor Instances	spark.executor.instances spark.dynamicAllocation.minExecutors	Initial number of executors run if dynamic allocation is enabled, with upper and lower bounds for the number of executors established.
	spark.dynamicAllocation.maxExecutors	
Shuffle Partitions	spark.sql.shuffle.partitions	Default number of partitions used when shuffling data for joins or aggregations.

**Table 15:** Effect on binning and training time for Spark’s configuration parameters.

Test ID	Executor Count	Executor Core Count	Total Executor Cores	Executor Memory	Total Executor Memory (GB)	Binning Time (seconds)	Training Time (seconds)
1	5	2	10	5	25	292.33	62.8
2	5	2	10	10	50	283.65	62.6
3	5	2	10	20	100	275.24	62.1
4	5	4	20	5	25	238.91	44.6
5	5	4	20	10	50	233.12	43.0
6	5	4	20	20	100	233.13	44.1
7	10	2	20	5	50	239.08	44.0
8	10	2	20	10	100	231.92	44.2
9	10	4	40	5	50	185.81	25.1
10	10	4	40	10	100	174.71	25.0
11	20	2	40	5	100	188.41	25.9
12	20	4	80	5	100	165.07	20.8
13	10	8	80	10	100	155.17	20.3
14	12	8	96	10	120	154.47	18.4
16	12	8	96	10	120	152.29	19.1
17	12	8	96	10	120	145.76	18.5
18	24	4	96	5	120	163.60	19.8
19	6	16	96	20	120	141.41	20.5
20	12	8	96	10	120	150.84	19.3
21	3	32	96	40	120	139.23	29.3
22	1	16	16	20	20	179.31	63.8
23	2	16	32	20	40	172.14	37.9
24	6	32	192	40	240	132.27	23.0
25	3	16	48	20	60	154.35	29.3

**Table 16:** Effect on total time with additional Spark configuration parameters.

Test ID	Executor Count	Cores Per Executor	Memory Per Executor	Total Exec. Cores	Total Executor Memory (GB)	Total Time (Seconds)	Shuffle Partitions	Driver Cores	Driver Memory (GB)
1	5	2	5	10	25	355.24	200	2	10
2	5	2	10	10	50	346.30	200	2	10
3	5	2	20	10	100	337.45	200	2	10
4	5	4	5	20	25	283.60	200	2	10
5	5	4	10	20	50	276.21	200	2	10
6	5	4	20	20	100	277.28	200	2	10
7	10	2	5	20	50	283.18	200	2	10
8	10	2	10	20	100	276.20	200	2	10
9	10	4	5	40	50	210.99	200	2	10
10	10	4	10	40	100	199.78	200	2	10
11	20	2	5	40	100	214.43	200	2	10
12	20	4	5	80	100	186.02	200	2	10
13	10	8	10	80	100	175.59	200	2	10
14	12	8	10	96	120	172.91	200	2	10
16	12	8	10	96	120	171.49	72	2	10
17	12	8	10	96	120	164.35	12	2	10
18	24	4	5	96	120	183.51	24	2	10
19	6	16	20	96	120	162.02	6	2	10
20	12	8	10	96	120	170.18	24	2	10
21	3	32	40	96	120	168.58	3	2	10
22	1	16	20	16	20	243.16	1	2	10
23	2	16	20	32	40	210.14	2	2	10
24	6	32	40	192	240	155.39	6	2	10
25	3	16	20	48	60	183.75	3	2	10
26	10	8	10	80	100	178.59	200	2	10
27	6	32	40	192	240	156.8	6	2	10
28	6	32	40	192	240	161.84	12	2	10
29	6	32	40	192	240	159.12	24	2	10
30	6	32	40	192	240	159.31	48	2	10
31	6	32	40	192	240	159.91	96	2	10
32	6	32	40	192	240	161.13	192	2	10
33	6	32	40	192	240	161.6	384	2	10
34	6	32	40	192	240	159.2	6	4	10
35	6	32	40	192	240	157.79	6	4	20
36	6	32	40	192	240	158.2	6	4	30

**Table 17:** Spark’s optimum configuration settings.

Config Setting	Value
Driver Cores	2
Driver Memory	10 g
Executor Instances	6
Executor Cores	6
Executor Memory	6
Shuffle Partitions	6

**Table 18:** Spark’s optimum resource settings and total allocation.

Resources	Total Allocation
Driver Cores	2
Driver Memory	10 g
Executor Total Cores	96
Executor Total Memory	120 g

**Table 19:** Reconnaissance: Performance of the various machine learning classifiers.

ML Algo.	Attr.	Accuracy	Precision	Recall	F-Measure	AUROC	FPR	Training	Testing
DT	6	99.30%	99.09%	98.58%	98.84%	99.10%	0.39%	27.933	0.087
DT	9	99.31%	99.10%	98.60%	98.85%	99.11%	0.39%	28.878	0.088
DT	12	99.35%	99.20%	98.65%	98.92%	99.15%	0.34%	29.75	0.086
DT	18	99.40%	99.69%	98.30%	98.99%	99.08%	0.13%	28.365	0.071
GBT	6	99.26%	99.39%	99.56%	99.48%	99.07%	1.42%	80.639	0.077
GBT	9	99.29%	99.39%	99.60%	99.50%	99.09%	1.42%	80.178	0.076
GBT	12	99.30%	99.38%	99.62%	99.50%	99.08%	1.46%	79.599	0.075
GBT	18	99.37%	99.23%	99.88%	99.55%	99.03%	1.81%	59.147	0.087
LR	6	96.52%	94.02%	94.38%	94.20%	95.91%	2.57%	22.1	0.057
LR	9	96.52%	94.02%	94.38%	94.20%	95.91%	2.57%	22.265	0.051
LR	12	96.52%	94.02%	94.38%	94.20%	95.91%	2.57%	22.372	0.051
LR	18	96.52%	94.02%	94.38%	94.20%	95.91%	2.57%	23.375	0.052
NB	6	95.84%	92.11%	94.19%	93.14%	95.37%	3.46%	15.634	0.053
NB	9	95.85%	92.11%	94.22%	93.15%	95.38%	3.46%	16.078	0.091
NB	12	95.85%	92.11%	94.21%	93.15%	95.38%	3.46%	15.7	0.062
NB	18	95.86%	92.12%	94.27%	93.18%	95.41%	3.46%	15.234	0.056
RF	6	99.19%	98.95%	99.90%	99.42%	98.72%	2.47%	56.257	0.048
RF	9	98.11%	97.39%	99.98%	98.67%	96.86%	6.26%	56.276	0.075
RF	12	99.19%	98.92%	99.94%	99.43%	98.70%	2.55%	56.473	0.052
RF	18	99.22%	98.94%	99.96%	99.45%	98.73%	2.51%	47.286	0.054
SVM	6	70.01%	0.00%	0.00%	0.00%	50.00%	0.00%	39.053	0.031
SVM	9	96.87%	95.23%	94.28%	94.75%	96.13%	2.02%	68.317	0.036
SVM	12	97.36%	97.08%	94.02%	95.53%	96.41%	1.21%	64.397	0.036
SVM	18	97.93%	99.04%	94.00%	96.45%	96.80%	0.39%	66.216	0.036

**Table 20:** Discovery: Performance of the various machine learning classifiers.

ML Algo.	Attr.	Accuracy	Precision	Recall	F-Measure	AUROC	FPR	Training	Testing
DT	6	99.81%	100.00%	99.33%	99.67%	99.67%	0.00%	3.798	0.057
DT	9	99.77%	100.00%	99.17%	99.58%	99.58%	0.00%	3.812	0.04
DT	12	99.91%	100.00%	99.67%	99.83%	99.83%	0.00%	4.091	0.08
DT	18	99.95%	100.00%	99.83%	99.92%	99.92%	0.00%	4.152	0.047
GBT	6	99.86%	99.80%	100.00%	99.90%	99.76%	0.49%	20.359	0.062
GBT	9	99.86%	99.80%	100.00%	99.90%	99.76%	0.49%	20.617	0.062
GBT	12	99.91%	99.87%	100.00%	99.93%	99.84%	0.32%	20.802	0.071
GBT	18	99.86%	99.87%	99.93%	99.90%	99.80%	0.32%	11.871	0.066
LR	6	97.89%	93.02%	100.00%	96.39%	98.53%	2.94%	4.997	0.04
LR	9	97.89%	93.02%	100.00%	96.39%	98.53%	2.94%	4.749	0.042
LR	12	97.89%	93.02%	100.00%	96.39%	98.53%	2.94%	4.746	0.041
LR	18	97.89%	93.02%	100.00%	96.39%	98.53%	2.94%	4.81	0.045
NB	6	97.28%	91.19%	100.00%	95.39%	98.10%	3.79%	2.975	0.041
NB	9	97.28%	91.19%	100.00%	95.39%	98.10%	3.79%	3.14	0.039
NB	12	94.55%	83.80%	100.00%	91.19%	96.21%	7.59%	3.144	0.038
NB	18	94.55%	83.80%	100.00%	91.19%	96.21%	7.59%	3.03	0.038
RF	6	99.62%	99.47%	100.00%	99.73%	99.35%	1.30%	13.171	0.062
RF	9	99.86%	99.80%	100.00%	99.90%	99.76%	0.49%	12.802	0.037
RF	12	99.86%	99.80%	100.00%	99.90%	99.76%	0.49%	12.689	0.039
RF	18	99.91%	99.87%	100.00%	99.93%	99.84%	0.32%	4.218	0.04
SVM	6	97.09%	91.51%	98.83%	95.03%	97.62%	3.60%	21.834	0.03
SVM	9	99.48%	100.00%	98.17%	99.07%	99.08%	0.00%	27.668	0.027
SVM	12	65.29%	31.85%	20.33%	24.82%	51.63%	17.07%	27.61	0.03
SVM	18	19.96%	0.00%	0.00%	0.00%	13.90%	72.20%	30.897	0.036

## Data Availability

The data are available at datasets.uwf.edu (accessed on 20 August 2020).

## Appendix A

**Table A1:** Attributes of Zeek Conn Logs Dataset.

Attribute Name	Description of Attribute
ts	Time of first packet
uid	Unique identifier of connection
id.orig_h	IP address of packet sender
id.orig_p	Outgoing port number
id.resp_h	IP address of packet receiver
id.resp_p	Incoming port number
proto	Transport layer protocol of connection
service	Identification of application protocol being sent over connection
duration	How long connection lasted
orig_bytes	Number of payload bytes originator sent
resp_bytes	Number of payload bytes responder sent
conn_state	Possible connection states
local_orig	If connection is responded to locally, value is T
local_resp	If connection is responded to locally, this value is T
missed_bytes	Number of bytes missed in content gaps, representative of packet loss
history	Records the state history of connections as a string of letter
orig_pkts	Number of packets originator sent. Set if: zeek:id:use_conn_size_analyzer = T
orig_ip_bytes	Number of packets responder sent. Set if: zeek:id:use_conn_size_analyzer = T
resp_pkts	Number of IP level bytes responder sent. Set if: zeek:id:use_conn_size_analyzer = T
resp_ip_bytes	Number of IP level bytes responder sent
community_id	
id	Connection’s 4-tuple of endpoint addresses/ports
tunnel_parents	If connection was over tunnel, indicates *uid* values for encapsulating parent(s) connections used over lifetime of inner connection
